# Total and differential WBC counts are related with coronary artery atherosclerosis and increase the risk for cardiovascular disease in Koreans

**DOI:** 10.1371/journal.pone.0180332

**Published:** 2017-07-28

**Authors:** Jung Hee Kim, Soo Lim, Kyong Soo Park, Hak Chul Jang, Sung Hee Choi

**Affiliations:** 1 Department of Internal Medicine, Seoul National University College of Medicine, Seoul, Korea; 2 Department of Internal Medicine, Seoul National University Bundang Hospital, Seongnam, Korea; Beijing Key Laboratory of Diabetes Prevention and Research, CHINA

## Abstract

**Objective:**

Inflammation is a key mechanism of atherosclerosis. White blood cells (WBCs) play a pivotal role in the inflammatory process. We investigated the relationships between total and differential WBC counts and multi-detector cardiac computed tomography (MDCT) findings, as well as the risk of cardiovascular disease in asymptomatic patients in Korea.

**Materials and methods:**

We recruited asymptomatic men (n = 7274) and women (n = 5478) aged ≥30 years who were free of known coronary heart disease. All patients underwent MDCT during a routine health check-up in the Seoul National University Bundang Hospital between 2006 and 2007, and were followed-up for 5.6 years. We reviewed medical records for cardiovascular diseases (CVDs) and covariates.

**Results:**

In covariate-adjusted logistic regression models for MDCT findings, subjects within the third tertile of all WBC subtypes had a higher risk for significant stenosis and noncalcified plaques compared with the first tertile of each subtype. In Cox proportional hazard regression models for the risk of CVDs, subjects within the third tertiles of lymphocytes and monocytes were at an increased risk of CVDs (total WBC, HR = 1.22 [1.02–1.44]; lymphocyte, HR = 1.47 [1.25–1.74]; monocytes, HR = 1.26 [1.02–1.35]) even after further adjustment for covariates and coronary artery stenosis.

**Conclusions:**

Total WBC counts were related with the severity of coronary artery disease, and higher WBC counts increased the risk of CVDs in asymptomatic Koreans mainly by virtue of monocytes.

## Introduction

Cardiovascular disease (CVD) is the leading cause of death in both Eastern and Western countries [1]. Although a number of risk factors contribute to the development of CVD, atherosclerosis has a pivotal role in its pathogenesis [2]. Moreover, a growing body of evidence supports a role for chronic inflammation in the atherosclerotic process [2, 3].

Interest in inflammation has prompted a search for additional biologic markers and particularly inflammatory markers such as C-reactive protein (CRP), interleukin-6, plasminogen activator inhibitor-1, and fibrinogen [4–6]. These markers can predict cardiovascular events or cardiovascular-related death. However, despite the interest in these novel inflammatory markers, white blood cells (WBCs) still have their traditional role in organizing the inflammatory process.

Several epidemiologic studies have shown that the WBC count is an independent risk factor of future cardiovascular event in patients without apparent CVD or who already have CVD [4, 7–13]. WBCs participate in the chronic inflammatory process and affect the development of CVD through multiple mechanisms that mediate inflammation, cause proteolytic and oxidative damage to the endothelial cells, block the microvasculature, induce hypercoagulability, and promote infarct expansion [14]. Although additional studies have investigated which subtype of WBCs (neutrophils, lymphocytes, or monocytes) play a major role in CVD, the results are inconsistent [15–17]. Moreover, since the discovery of new biomarkers, the diagnostic and prognostic utility of WBCs in CVD is widely unappreciated. Notwithstanding the declining interest, the measurement of WBCs is easy, inexpensive, and noninvasive. Therefore, further studies are needed to assess the true impact of leukocytosis in coronary heart disease, compare it with other inflammatory markers such as CRP, and promote its use in predicting coronary heart disease.

With advancements in imaging technology, multi-detector cardiac computed tomography (MDCT) enables nearly motion-free visualization of the coronary arteries and accurate detection of significant stenosis, without invasive coronary artery angiography [18]. Using MDCT, we noninvasively assessed the extent of coronary artery stenosis, coronary artery calcification score (CACS), and atherosclerotic plaque morphology in previous studies [18, 19]. As a result, it has been used as a screening test for coronary artery disease and can predict cardiovascular events, despite the limited utility of MDCT owing to radiation exposure [19].

Total and differential WBC counts might mediate the inflammatory process and be related with coronary artery stenosis, coronary artery calcification, and coronary plaque even in asymptomatic subjects and ultimately result in the development of CVD. Therefore, we assessed the relationships between total and differential WBC counts and subclinical coronary artery sclerosis, as measured using MDCT, in a cross-sectional study and the longitudinal risk of CVD based on WBC counts in asymptomatic Koreans.

## Materials and methods

### Subjects

We recruited 7,401 men and 5,586 women aged ≥30 years who were asymptomatic and free of known coronary heart disease from the Seoul National University Bundang Hospital between 2006 and 2007. All patients underwent an MDCT as part of a routine health check-up after they agreed to participate in the study and had been informed of the possible risks of undergoing a CT. The details of recruitment have been described previously [20]. This study was approved by the institutional review board of the Seoul National University Bundang Hospital; the need for consent was waived.

#### Measurement of anthropometric and biochemical parameters

Height and body weight were measured to the nearest 0.1 cm and 0.1 kg, respectively. Body mass index (BMI) was calculated as weight divided by height (in meters) squared. Blood pressure was measured three times between 7 AM and 9 AM after the patient sat in a relaxed state for at least 10 minutes.

After a 12-hour overnight fast, blood samples were drawn from the antecubital vein. Plasma was separated immediately with centrifugation (2000 rpm for 20 minutes at 4°C), and biochemical measurements were obtained within 2 hours. A complete blood cell count analysis including total and differential WBC counts, was performed using an XE-2100 (Sysmex, Kobe, Japan). The fasting plasma concentrations of glucose, total cholesterol, triglycerides, high-density lipoprotein (HDL) cholesterol, and low-density lipoprotein (LDL) cholesterol were measured enzymatically with a Hitachi 747 chemical analyzer (Hitachi, Tokyo, Japan). Hemoglobin A1c level was measured with an immunoturbidimetric assay performed with a Cobra Integra 800 automatic analyzer (Roche Diagnostics, Basel, Switzerland). Serum high-sensitivity CRP (hsCRP) levels were measured with a high-sensitivity automated immunoturbidimetric method (CRP II Latex ×2; Denka Seiken, Tokyo, Japan).

Smoking status was defined as follows: current smoker, patient currently smoked and had smoked for at least 1 year; nonsmoker, patient had never smoked; or ex-smoker, patient smoked but quit. Type 2 diabetes was defined as a fasting plasma glucose level ≥126 mg/dL or current use of antidiabetic treatment. Hypertension was defined as two consecutive systolic/diastolic blood pressure measurements >140/90 mmHg or current use of antihypertensive medication. Dyslipidemia was defined as low HDL cholesterol (<40 mg/dL for men, <50 mg/dL for women), high triglyceride level ≥ 150 mg/dL or taking lipid-lowering agents.

#### Multidetector cardiac computed tomography (MDCT) data acquisition

Subjects with a heart rate >70 beats per minute were administered 10–30 mg intravenous esmolol (Brevibloc; Jeil Pharmaceutical, Seoul, Korea) before the MDCT. With the exception of patients with contraindications to nitroglycerin, 0.6 mg of nitroglycerine was immediately administered sublingually before contrast material injection [21]. In all patients, CT angiography was performed with a 64–detector row CT scanner (Brilliance 64; Philips Medical Systems, Best, the Netherlands) with 643 0.625-mm section collimation and 420-msec rotation time. Scanning for the CACS was performed with 120-kV tube voltage, 220-mA tube current, and 2.5-mm section thickness. CT angiography was performed with 120-kV tube voltage and 800-mA tube current with electrocardiographically gated dose modulation. An 80-mL bolus of iomeprol (Iomeron 400; Bracco, Milan, Italy) was intravenously injected at a rate of 4 mL/sec, followed by a 50-mL saline chaser. Images were initially reconstructed in the mid-diastolic phase of the cardiac cycle (75% of the RR interval). If motion artifacts were observed, additional reconstructions were performed at other cardiac phases during retrospectively gated helical acquisitions. The CACS was calculated with the Agaston score using a threshold of 130 Hounsfield Units (HUs) on precontrast images [22].

#### Multidetector cardiac computed tomography (MDCT) image analysis

All images were analyzed independently in a blinded fashion by two experienced radiologists using a three-dimensional workstation (Brilliance; Philips Medical Systems). After the independent evaluations, a consensus interpretation regarding the final MDCT diagnosis was reached. Each lesion was identified using a multiplanar reconstruction technique and maximum intensity projection of the short-axis and two- and four-chamber views. We analyzed the plaque characteristics on a per-segment basis according to the modified American Heart Association classification [23]. All coronary segments >1.5 mm in diameter were assessed. Image quality was evaluated on a per-segment basis using a four-point grading scale (1, absence of any artifacts; 2, slight artifacts but fully evaluable; 3, artifacts but evaluable; 4, noninterpretable). A segment with noninterpretable image quality was not included in the analysis. Thereafter, the interpretable segments were evaluated for plaque severity and characteristics. Plaques were identified as structures >1 mm within or adjacent to the vessel lumen, which could be clearly distinguished from the lumen and surrounding epicardial fat.

We evaluated the degree of stenosis, plaque type, and coronary artery calcification. To determine the number of atherosclerotic coronary segments, coronary segments with any plaque were included. The coronary artery stenosis was estimated when the contrast material-enhanced portion of the coronary lumen was semiautomatically traced at the maximal stenotic site and compared with the mean value for the proximal and distal reference sites [24]. Stenosis >50% was defined as significant. Plaque type was classified as follows: calcified, presence of calcified tissue that comprised >50% of the plaque area (attenuation >130 HU) on native images, or non-calcified, presence of plaques with <50% calcium in the plaque area [24]. Significant coronary artery calcification was defined as a CACS ≥100.

#### Cardiovascular disease coding

During the follow-up duration of 5.6 (5.58–5.70) years, we identified subjects with CVD according to the International Classification of Diseases: angina pectoris (I209), acute myocardial infarction (I219), coronary atherosclerosis (I2519), coronary disease in diabetes mellitus (E1458), old myocardial infarction (I252), stable angina (I2088), unstable angina (I200), variant angina (I201), acute anterior wall myocardial infarction (I210), acute inferior wall myocardial infarction (I211), acute lateral/posterior wall myocardial infarction (I212), acute subendocardial myocardial infarction (I2149), and complication following acute myocardial infarction (I238).

### Statistical analysis

All data are expressed as the mean ± standard deviation. Baseline characteristics and multidetector CT findings were compared using the Student t test or χ^2^ test. Multivariable logistic regression analyses were performed for MDCT findings according to tertiles of the total WBC count, neutrophil count, lymphocyte count, and monocyte count. We present the unadjusted and adjusted odds ratios (ORs) for the MDCT findings of significant coronary artery stenosis, noncalcified plaque, and CACS, according to the third *vs*. first WBC, neutrophil, lymphocyte, and monocyte tertiles, with age, sex, BMI, smoking status, diabetes, hypertension, and dyslipidemia as covariates. Cox proportional hazard regression models were used to analyze the risk of CVD according to the total and differential WBC count tertiles: model 1, unadjusted hazard ratios (HR) for the top to bottom tertiles; model 2, HRs adjusted for age, sex, BMI, smoking status, diabetes, hypertension, and dyslipidemia; and model 3, HRs also adjusted for significant coronary artery stenosis. We present the Kaplan-Meier curves for cumulative CVD-free survival according to the WBC count tertiles Receiver operating characteristics (ROC) curve analyses were performed to examine which WBC subtype has the highest predictive value for CVD independent of total WBC counts. Statistical significance was defined as *P* < .05. All analyses were performed with SPSS, version 16.0 (SPSS Inc., Chicago, IL).

## Results

Table 1 shows the descriptive characteristics of the study population. The mean age of men and women were 53.8 ± 12.1 and 56.4 ± 12.0 years, respectively. Men had greater BMIs than women. Total and differential WBC counts were slightly higher in men than in women. Serum triglyceride and LDL cholesterol levels were higher, but serum HDL cholesterol levels were lower, in men than in women. Diabetes, hypertension, and >50% coronary artery stenosis were more prevalent in men than in women.

**Table 1:** Descriptive characteristics of study population.

	Men (n = 7,401)	Women (n = 5,586)	*p* value
Age (years)	53.8 ± 12.1	56.4 ± 12.0	< 0.001
Body mass index (kg/m^2^)	25.2 ± 2.9	24.5 ± 3.4	< 0.001
White blood cell (/mm^3^)	6106.0 ± 1470.6	5528.0 ± 1462.2	< 0.001
Neutrophil (/mm^3^)	3370.0 ±1118.4	3104.5 ± 1154.8	< 0.001
Lymphocyte (/mm^3^)	2103.3 ± 614.4	1932.6 ± 594.4	< 0.001
Monocyte (/mm^3^)	394.4 ± 142.6	329.4 ± 121.8	< 0.001
Fasting plasma glucose (mg/dL)	120.2 ± 37.5	118.5 ± 38.1	0.293
HbA1c	5.70 (5.40–6.00)	5.60 (5.35–6.00)	<0.001
Total cholesterol (mg/dL)	200.4 ± 36.6	202.5 ± 38.0	0.001
Triglycerides (mg/dL)	150.6 ± 96.8	113.1 ± 77.7	<0.001
HDL-cholesterol (mg/dL)	51.3 ± 12.4	59.2 ± 14.4	< 0.001
LDL-cholesterol (mg/dL)	109.3 ± 26.5	107.8 ± 28.0	0.003
hsCRP	0.01 (0.01–0.19)	0.01 (0.01–0.14)	<0.001
Diabetes	1282 (17.7%)	712 (13.1%)	<0.001
Hypertension	2735 (37.6%)	1721 (31.4%)	<0.001
Lipid-lowering agents	1076 (14.5%)	665 (11.9%)	<0.001
Coronary artery calcium scores	50.1 ± 243.5	27.1 ± 138.9	<0.001
>50% stenosis	661 (9.1%)	261 (4.8%)	<0.001
Plaque			<0.001
Calcified	2104 (28.4%)	895 (16.0%)	
Non-calcified	5297 (71.6%)	4691 (84.0%)	
Multiple vessel involvement	202 (2.8%)	74 (1.4%)	<0.001

Data area shown as mean ± standard deviation (SD), median (interquantile range) or n (%). HbA1C, glycated hemoglobin; HDL, high-density lipoprotein; LDL, low-density lipoprotein; hsCRP, highly sensitive C-reactive protein

Total and differential WBC counts increased with age and BMI (Table 2). All subtypes of WBC were positively associated with serum triglyceride levels and negatively associated with serum HDL cholesterol levels. hsCRP was positively related with all WBC subtypes. Correlation analyses between WBC subtypes and metabolic parameters did not show that any of the subtypes (neutrophils, lymphocytes, and monocytes) were dominant. In the partial correlation analysis after adjusted for age, gender, BMI, smoking, diabetes, hypertension, and dyslipidemia, the results were similar to the unadjusted correlation analyses (data not shown).

**Table 2:** Correlation between WBC subtypes and metabolic parameters.

	WBC	Neutrophil	Lymphocyte	Monocyte
Age	0.080*	0.060*	0.027*	0.133*
Body mass index	0.125*	0.042*	0.217*	0.069*
Fasting plasma glucose	0.130*	0.096*	0.088*	0.122*
HbA1c	0.213*	0.158*	0.174*	0.184*
Total cholesterol	0.047*	0.008	0.124*	-0.032*
Triglyceride	0.290*	0.193*	0.286*	0.206*
HDL cholesterol	-0.229*	-0.179*	-0.164*	-0.201*
LDL cholesterol	0.063*	0.026*	0.117*	-0.005
hsCRP	0.251*	0.239*	0.045*	0.271*

*, *p* value < .05; HbA1C, glycated hemoglobin; HDL, high-density lipoprotein; LDL, low-density lipoprotein; hsCRP, highly sensitive C-reactive protein

**Fig 1:**
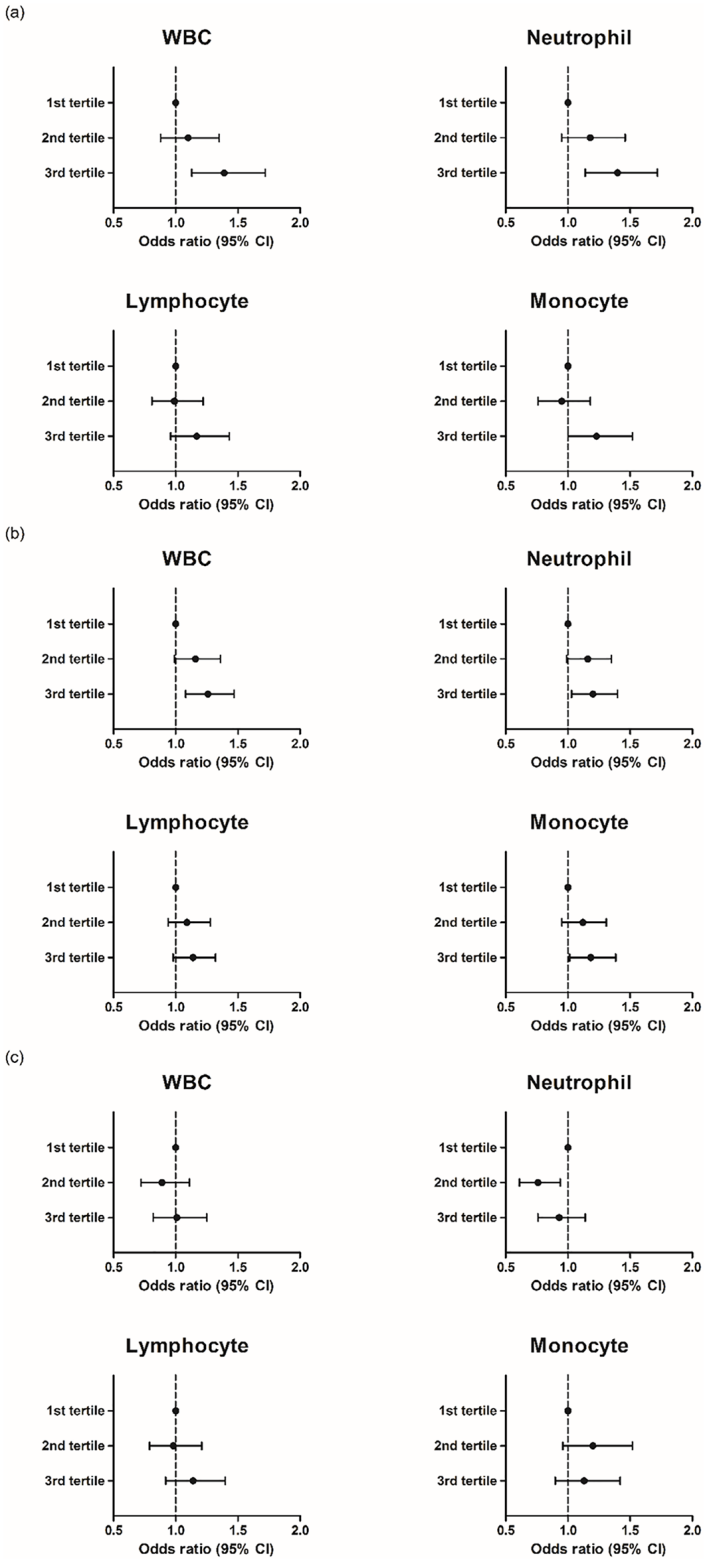
Logistic regression models for MDCT findings: (a) coronary artery stenosis >50%, (b) non-calcified plaque (c) coronary artery calcium score > 100) according to tertiles of total and differential WBC counts. (Data are shown as odds ratios (95% CI) adjusted for age, gender, BMI, smoking, diabetes, hypertension, and dyslipiemia).

According to the logistic regression models for MDCT findings, subjects within the highest tertile of each WBC subtype had higher risks of significant stenosis and noncalcified plaques compared to subjects within the lowest tertile, after adjustment for age, sex, BMI, smoking, diabetes, hypertension, and dyslipidemia. However, higher total and differential WBC counts did not increase the risk for CACS >100 (Fig 1).

**Fig 2:**
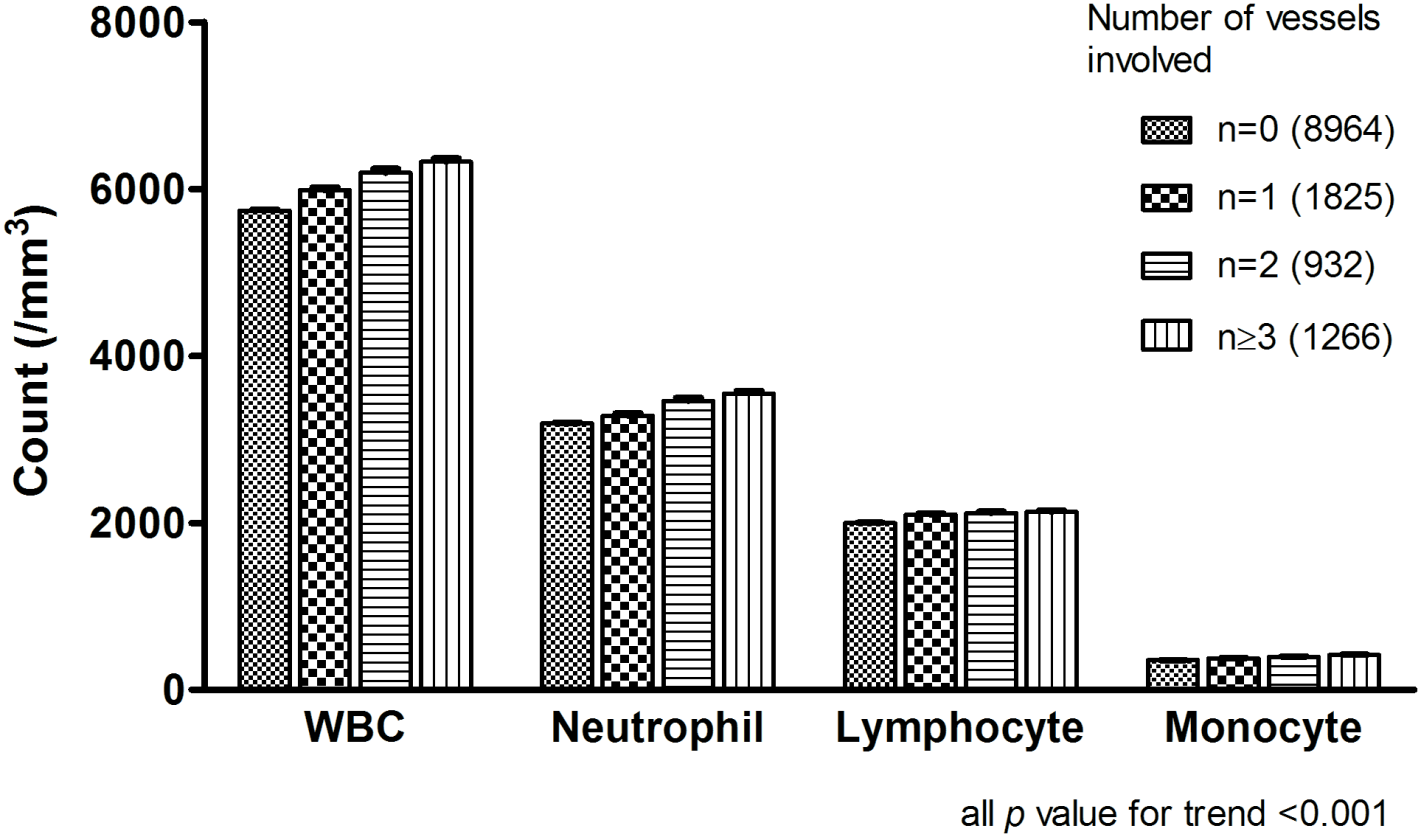
The number of each WBC subtype according to the number of vessels involved. (all *p* value for trend <0.001).

We also explored the number of each WBC subtype according to the number of vessels involved (Fig 2). We confirmed the dose-dependent effect of all WBC subtypes on the number of vessels involved.

In model 1 of the Cox proportional hazard regression analyses, subjects within the third tertile of all WBC subtypes had a higher risk for CVD than those within the first tertile (Table 3).

**Table 3:** Cox proportional hazard regression analyses for cardiovascular disease according to tertiles of total and differential WBC counts.

	WBC	Neutrophil	Lymphocyte	Monocyte
Model 1	1.73 (1.48–2.02)	1.30 (1.11–1.51)	1.50 (1.29–1.75)	2.01 (1.71–2.36)
Model 2	1.24 (1.04–1.47)	0.95 (0.80–1.12)	1.55 (1.31–1.83)	1.28 (1.07–1.52)
Model 3	1.22 (1.02–1.44)	0.87 (0.73–1.03)	1.47 (1.25–1.74)	1.26 (1.02–1.35)

Data are shown as hazard ratios (HR) of the top to bottom tertile (95% CI); Model1: unadjusted; Model2: adjusted for age, gender, BMI, smoking, diabetes, hypertension and dyslipidemia; Model3: additionally adjusted for significant coronary artery stenosis

**Fig 3:**
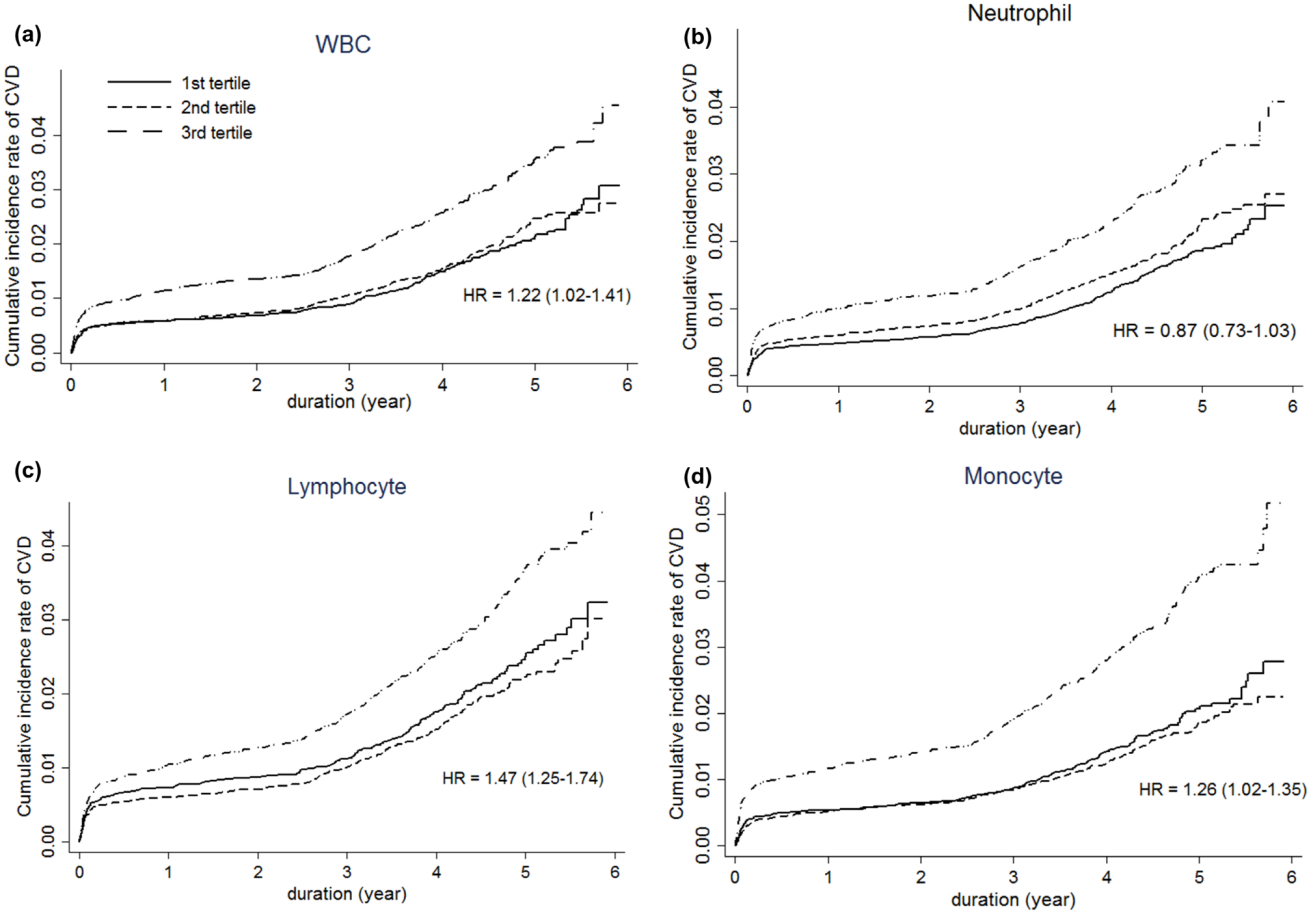
Cumulative Incidence rate of cardiovascular disease according to tertiles of (a) WBC, (b) Neutrophil, (c) Lymphocyte and (d) Monocyte. (Data are shown as hazard ratios (HR) of the top to bottom tertile (95% CI); adjusted for age, gender, BMI, smoking, diabetes, hypertension, dyslipidemia and significant stenosis) (median (years): 5.60 (5.58–5.70)).

However, neutrophil counts were no longer significant in model 2, after adjustment for covariates such as age, sex, BMI, smoking, diabetes, hypertension, dyslipidemia. After additionally adjusting for significant coronary artery stenosis in model 3, subjects within the third tertiles of lymphocytes and monocytes were at an increased risk of CVD (total WBC, HR = 1.22 [1.02–1.44]; lymphocyte, HR = 1.47[1.25–1.74]; monocytes, HR = 1.26[1.02–1.35]) (Fig 3). Therefore, total WBC counts independently increased the risk of CVD, mainly by virtue of lymphocytes and monocytes.

We further conducted ROC curve analyses to examine which WBC subtype has the highest predictive value for CVD (Table 4). Neutrophils had a lower AUC value than total WBC. The AUC of lymphocytes was similar to total WBC. The AUC of monocytes was higher than that of total WBC.

**Table 4:** Receiver operating characteristics (ROC) curve analyses of each WBC subtypes for cardiovascular disease.

	AUC	95% CI	*p* values compared with WBC
WBC	0.586	0.571–0.601	
Neutrophil	0.554	0.539–0.570	<0.001
Lymphocyte	0.583	0.567–0.599	0.691
Monocyte	0.613	0.598–0.628	<0.001

AUC, area under the curve; CI, confidence interval

## Discussion

In the present study, all differential WBC counts were significantly related with fasting glucose, triglyceride, HDL cholesterol, and hsCRP levels. On MDCT, total WBCs and all WBC subtypes increased the risk for significant coronary artery stenosis and noncalcified plaque even after adjusting for age, sex, BMI, smoking status, diabetes, hypertension, and dyslipidemia. However, only subjects within the highest tertiles of total WBC, lymphocyte, and monocyte counts were at a higher risk for developing CVD.

There have been few studies regarding the direct relationship between WBC counts and coronary artery status using MDCT findings in subjects free of CVD. Previous studies have shown that angiographically driven coronary artery stenosis is related to WBC count [8, 25, 26]. The MDCT showed not only luminal narrowing but also coronary artery calcification and characteristics of plaque at the coronary artery wall that is not possible with conventional coronary angiography. In the only study regarding the association between total WBC count and coronary artery stenosis or plaque characteristics on MDCT of 817 patients at high risk of CVD [27]. Total WBC counts, in addition to other well-known cardiovascular risk factors, were independently associated with the presence and severity of coronary atherosclerosis, and plaque type was not associated with total WBC count [27]. However, non-calcified coronary artery plaques detected by MDCT are associated with acute coronary syndrome and are considered more vulnerable than calcified plaques [28, 29]. In the present study, which was conducted with a large number of subclinical subjects without CVD, significant coronary artery stenosis was related with all WBC subtypes, in addition to other cardiovascular risk factors. In addition, only non-calcified plaque was associated with neutrophils, lymphocytes, monocytes, and the total WBC count. To the best of our knowledge, this is the first study to evaluate the relationship between WBC subtypes and coronary artery status on MDCT in asymptomatic subjects.

Epidemiological studies have shown a positive correlation between WBC count and risk of CVD both in healthy subjects free of CVD and in patients with CVD [7, 9, 11–13, 26, 30]. In the NHANES I Epidemiologic Follow-up Study, compared with WBC counts <6600 cells/mm^3^, WBC counts >8100 cells/mm^3^ were associated with an increased risk of coronary heart disease in white men (relative risk [RR] = 1.31) and white women (RR = 1.31) aged 45–74 years, after adjusting for baseline risk factors.[30]. In a meta-analysis of the 7 largest studies of the associations between total WBC count and coronary heart disease, accounting for 5337 participants with coronary heart disease, a difference in WBC of 2800 cells/mm^3^ was associated with a combined RR of 1.4 [4]. Similarly, total WBC count increased the risk for future CVD in the present study, after adjusting for traditional cardiovascular risk factors.

The respective influence of WBC subtypes on CVD in subjects free of CVD has yet to be determined [14–17, 31]. In the EPIC-Norfolk Prospective Population Study, the higher risk for CVD associated with increased total WBC count seemed to be accounted for by the increased neutrophil count, but not lymphocyte or monocyte count [32]. In a meta-analysis comprising a total of 1764 incident cases of coronary artery disease, the association between CVD and neutrophil counts was somewhat stronger than the associations with other specific WBC subtypes (combined RR = 1.33 [1.17–1.50]) [16]. However, monocytes have been implicated as one of the WBC types associated with CVDs [8, 31]. Monocytes are recruited from the peripheral blood into the vessel wall after endothelial injury. The recruited monocytes differentiate into macrophages that phagocytose lipids and secrete metalloproteinase enzymes and reactive oxygen species within the atherosclerotic lesion [3, 8, 33]. The Paris Prospective Study II revealed that the adjusted risk of CVD increased 1.15 times for each 100-cell/mm^3^ increase in monocyte count [31]. The role of lymphocytes on CVDs seems to be weak, but in a meta-analysis regarding the lymphocyte count and incidence of diabetes, the top tertile of lymphocytes was 1.26 times higher than the bottom tertile [34]. In a previous study from the United Kingdom, the age-adjusted relative odds of CVD were highest for men with the highest neutrophil, lymphocyte, or monocyte count [35]. In contrast, lymphocytes and monocytes were stronger predictors than neutrophils in the present study. In further analyses using ROC curve, monocyte had the highest predictive value for CVD regardless of total WBC counts while lymphocyte was dependent on total WBC count. The lack of significance of neutrophils on the incidence of CVDs may be attributed to the complex interaction between neutrophils and smoking, diabetes, hypertension, or dyslipidemia. In the analysis of the effect of WBC subtype on CVDs, when adjusted for significant coronary artery stenosis, lymphocyte and monocyte counts predicted CVD independent of coronary artery stenosis, despite the significant role of coronary artery stenosis for cardiovascular events [19, 29]. Although the relationship between WBC subtypes and MDCT findings or CVD was different than that reported previously, the supplementary role of lymphocytes and monocytes explains the discrepancy. Furthermore, the association between the MDCT findings and WBC was evaluated in a cross-sectional manner but the risk for CVD was reviewed longitudinally. To the best of our knowledge, there have also been no studies simultaneously analyzing the relationship between WBC subtypes and CVD or MDCT findings.

The present study had several limitations. First, because a retrospective, cross-sectional review of data was performed, causal relationships could not be determined; instead, we were only able to describe the phenomenon. Second, the study was performed in a single center with asymptomatic Korean subjects who had no apparent CVD, which could result in selection bias. However, the sample size was large enough to overcome this bias. Third, we included only asymptomatic individuals without a history of CVD, limiting the generalization of the findings to symptomatic individuals. This also contributed to the delayed onset of cardiovascular diseases. Fourth, the WBC count was measured only once, *i*.*e*., at the time of the MDCT, which may reduce the accuracy of the average WBC count for each individual. However, to avoid inaccurate WBC counts, we excluded subjects with a WBC count >10,000 cells/mm^3^.

## Conclusions

Chronic inflammation is a key feature of atherosclerosis, and WBC count is a marker of inflammation that is widely available in clinical practice. A high WBC count, regardless of the subtype, is associated with non-calcified plaques well as significant coronary artery stenosis on MDCT in asymptomatic individuals. Moreover, WBC counts, especially monocytes, were independent risk factors of CVDs. Thus, WBC could be a readily available and informative marker for CVDs in asymptomatic individuals. Further research is required to determine which WBC subtype is more important for mediating atherosclerosis.
